# Dicarbonyl­{3,3′-di-*tert*-butyl-5,5′-di­meth­oxy-2,2′-bis­[(4,4,5,5-tetra­phenyl-1,3,2-dioxa­phospho­lan-2-yl)­oxy-κ*P*]biphen­yl}hydridorhodium(I) diethyl ether monosolvate

**DOI:** 10.1107/S1600536812011786

**Published:** 2012-03-28

**Authors:** Detlef Selent, Anke Spannenberg, Armin Börner

**Affiliations:** a Leibniz-Institut für Katalyse e. V. an der Universität Rostock, Albert-Einstein-Strasse 29a, 18059 Rostock, Germany

## Abstract

In the title compound, [Rh(C_74_H_68_O_8_P_2_)H(CO)_2_]·C_4_H_10_O, the C_2_HP_2_ coordination set at the Rh^I^ ion is arranged in a distorted trigonal–planar geometry with one P atom of the diphosphite mol­ecule and the H atom adopting the axial coordination sites.

## Related literature

For another crystal structure of a dicarbonyl hydrido complex of rhodium(I), see: Van Rooy *et al.* (1995 ▶, 1996 ▶). The title compound has recently been studied in solution, see: Selent *et al.* (2011 ▶). Structural information on this labile compound class is usually obtained by spectroscopy, see, for example: Dieleman *et al.* (2001 ▶); Axet *et al.* (2007 ▶).
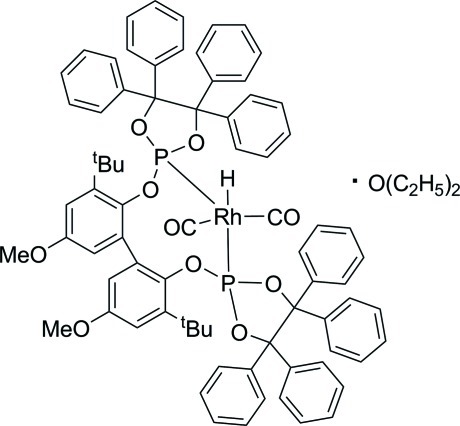


## Experimental

### 

#### Crystal data

[Rh(C_74_H_68_O_8_P_2_)H(CO)_2_]·C_4_H_10_O
*M* *_r_* = 1381.28
Monoclinic, 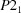

*a* = 11.1489 (2) Å
*b* = 25.8458 (4) Å
*c* = 12.5941 (3) Å
β = 110.263 (2)°
*V* = 3404.43 (11) Å^3^
*Z* = 2
Mo *K*α radiation
μ = 0.36 mm^−1^
*T* = 200 K
0.40 × 0.35 × 0.15 mm

#### Data collection

Stoe IPDS II diffractometer
Absorption correction: numerical (*X-SHAPE* and *X-RED32*; Stoe & Cie, 2005 ▶) *T*_min_ = 0.835, *T*_max_ = 0.959
59171 measured reflections
16262 independent reflections
14238 reflections with *I* > 2σ(*I*)
*R*_int_ = 0.026

#### Refinement

*R*[*F* ^2^ > 2σ(*F* ^2^)] = 0.028
*wR*(*F* ^2^) = 0.063
*S* = 0.94
16262 reflections
834 parameters
35 restraints
H atoms treated by a mixture of independent and constrained refinement
Δρ_max_ = 0.66 e Å^−3^
Δρ_min_ = −0.47 e Å^−3^
Absolute structure: Flack (1983 ▶), 7944 Friedel pairs
Flack parameter: −0.026 (11)

### 

Data collection: *X-AREA* (Stoe & Cie, 2005 ▶); cell refinement: *X-AREA*; data reduction: *X-AREA*; program(s) used to solve structure: *SHELXS97* (Sheldrick, 2008 ▶); program(s) used to refine structure: *SHELXL97* (Sheldrick, 2008 ▶); molecular graphics: *XP* in *SHELXTL* (Sheldrick, 2008 ▶); software used to prepare material for publication: *SHELXTL*.

## Supplementary Material

Crystal structure: contains datablock(s) I, global. DOI: 10.1107/S1600536812011786/bt5848sup1.cif

Structure factors: contains datablock(s) I. DOI: 10.1107/S1600536812011786/bt5848Isup2.hkl

Additional supplementary materials:  crystallographic information; 3D view; checkCIF report

## Figures and Tables

**Fig. 1.**
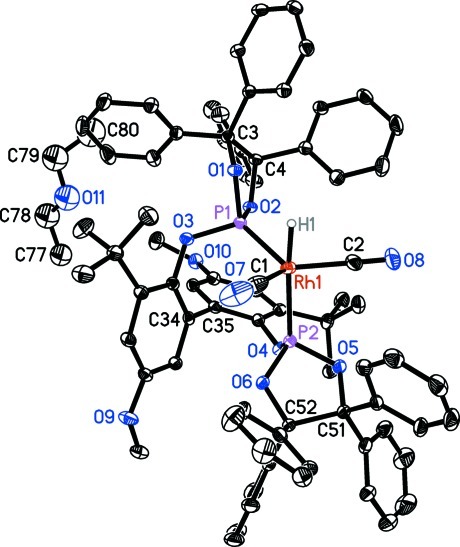
The molecular structure of the title compound; displacement ellipsoids are drawn at the 30% probability level. Hydrogen atoms except H1 are omitted for clarity.

## supplementary crystallographic information

### Comment

The reaction of a mixture of dicarbonyl(acetylacetonato-κ*O,O'*)rhodium(I) and 3,3'-di-*tert*-butyl-5,5'-dimethoxy-2,2'-bis[(4,4,5,5-tetraphenyl-1,3,2-dioxaphospholan-2-yl)oxy]biphenyl under an atmosphere of hydrogen and carbon monoxide in toluene affords the elimination of acetylacetone and the formation of the rhodium hydrido complex which, after recrystallization, gives the title compound (figure 1). The distances Rh1—P1 = 2.3045 (5) Å and Rh1—P2 = 2.2913 (5) Å and the angle P1—Rh1—P2 = 109.66 (2)° do significantly differ from those (Rh1—P1 = 2.255 (3), Rh1—P2 = 2.239 (3) Å, P1—Rh1—P2 = 115.95 (9)°) found in the only molecular structure of a similar rhodium complex known to date (Van Rooy *et al.* 1995, 1996). In the solid state, the phosphorus atoms of the diphosphite molecule adopt formally different axial and equatorial sites of the idealized trigonal bipyramidal geometry around the rhodium center which is in contrast to the bisequatorial arrangement determined for both, the solution and the theoretical gas phase structure (Selent *et al.*, 2011). The hydride could be found from difference Fourier map; the Rh1—H1 distance was refined to 1.42 (3) Å.

### Experimental

A mixture of dicarbonyl(acetylacetonato-κ*O,O'*)rhodium(I) (0.5161 g, 2 mmol) and 3,3'-di-*tert*-butyl-5,5'-dimethoxy-2,2'-bis[(4,4,5,5-tetraphenyl-1,3,2-dioxaphospholan-2-yl)oxy]biphenyl (2.524 g, 2.2 mmol) was dissolved in toluene (50 ml). The resulting solution was transferred to an autoclave and then stirred under an atmosphere of carbon monoxide and hydrogen (1:1, 2.0 MPa) at 70°C for 2 h. The clear, pale yellow solution obtained after cooling and depressurization was evaporated to dryness *in vacuo*. The residue was crystallized from diethyl ether to give 2.108 g (1.613 mmol, 81%) of the title compound. ^1^H-NMR(toluene-D_8_): -10.00 (d, ^1^*J*_HRh_ = 3.4 Hz) p.p.m.. ^31^P-NMR (toluene-D_8_): 166.1 (d, ^1^*J*_PRh_ = 235 Hz) p.p.m.. Elemental analysis (calc. for C_76_H_69_O_10_P_2_Rh = 1307.229 g/mol): C, 69.75 (69.83); H, 5.48 (5.32); P, 4.56 (4.74); Rh, 7.73(7.87) %. ESI-TOF/HRMS: *m/e* 1317.3097 (M—CO+K)^+^, 1249.34397 (*M*-2CO)^+^. Crystals suitable for X-ray analysis were obtained by recrystallization from diethyl ether.

### Refinement

H1 was found from difference Fourier map and refined freely. All other H atoms were placed in idealized positions with d(C—H) = 0.95 Å (CH), 0.99 Å (CH_2_) and 0.98 Å (CH_3_) and refined using a riding model with *U*_iso_(H) fixed at 1.2 *U*_eq_(C) for CH, CH_2_ and 1.5 *U*_eq_(C) for CH_3_. The solvent atoms were only isotropically refined.

### Crystal data

**Table:**

[Rh(C_74_H_68_O_8_P_2_)H(CO)_2_]·C_4_H_10_O	*F*(000) = 1444
*M**_r_* = 1381.28	*D*_x_ = 1.347 Mg m^−^^3^
Monoclinic, *P*2_1_	Mo *K*α radiation, λ = 0.71073 Å
*a* = 11.1489 (2) Å	Cell parameters from 15841 reflections
*b* = 25.8458 (4) Å	θ = 1.7–28.4°
*c* = 12.5941 (3) Å	µ = 0.36 mm^−^^1^
β = 110.263 (2)°	*T* = 200 K
*V* = 3404.43 (11) Å^3^	Prism, colourless
*Z* = 2	0.40 × 0.35 × 0.15 mm

### Data collection

**Table:**

Stoe IPDS II diffractometer	16262 independent reflections
Radiation source: fine-focus sealed tube	14238 reflections with *I* > 2σ(*I*)
Graphite monochromator	*R*_int_ = 0.026
ω scans	θ_max_ = 27.9°, θ_min_ = 1.6°
Absorption correction: numerical (*X-SHAPE* and *X-RED32*; Stoe & Cie, 2005)	*h* = −14→14
*T*_min_ = 0.835, *T*_max_ = 0.959	*k* = −34→34
59171 measured reflections	*l* = −16→16

### Refinement

**Table:**

Refinement on *F*^2^	Secondary atom site location: difference Fourier map
Least-squares matrix: full	Hydrogen site location: inferred from neighbouring sites
*R*[*F*^2^ > 2σ(*F*^2^)] = 0.028	H atoms treated by a mixture of independent and constrained refinement
*wR*(*F*^2^) = 0.063	*w* = 1/[σ^2^(*F*_o_^2^) + (0.0377*P*)^2^] where *P* = (*F*_o_^2^ + 2*F*_c_^2^)/3
*S* = 0.94	(Δ/σ)_max_ = 0.001
16262 reflections	Δρ_max_ = 0.66 e Å^−^^3^
834 parameters	Δρ_min_ = −0.47 e Å^−^^3^
35 restraints	Absolute structure: Flack (1983), 7944 Friedel pairs
Primary atom site location: structure-invariant direct methods	Flack parameter: −0.026 (11)

### Special details

**Table:**

Geometry. All e.s.d.'s (except the e.s.d. in the dihedral angle between two l.s. planes) are estimated using the full covariance matrix. The cell e.s.d.'s are taken into account individually in the estimation of e.s.d.'s in distances, angles and torsion angles; correlations between e.s.d.'s in cell parameters are only used when they are defined by crystal symmetry. An approximate (isotropic) treatment of cell e.s.d.'s is used for estimating e.s.d.'s involving l.s. planes.
Refinement. Refinement of *F*^2^ against ALL reflections. The weighted *R*-factor *wR* and goodness of fit *S* are based on *F*^2^, conventional *R*-factors *R* are based on *F*, with *F* set to zero for negative *F*^2^. The threshold expression of *F*^2^ > σ(*F*^2^) is used only for calculating *R*-factors(gt) *etc*. and is not relevant to the choice of reflections for refinement. *R*-factors based on *F*^2^ are statistically about twice as large as those based on *F*, and *R*- factors based on ALL data will be even larger.

### Fractional atomic coordinates and isotropic or equivalent isotropic displacement parameters (Å^2^)

**Table:**

	*x*	*y*	*z*	*U*_iso_*/*U*_eq_	
C77	1.0804 (4)	0.91333 (17)	0.5094 (3)	0.0878 (11)*	
H77A	1.1508	0.9343	0.5030	0.132*	
H77B	1.1042	0.8767	0.5147	0.132*	
H77C	1.0626	0.9236	0.5774	0.132*	
C78	0.9699 (5)	0.9212 (2)	0.4129 (4)	0.1113 (15)*	
H78A	0.9490	0.9586	0.4077	0.134*	
H78B	0.9908	0.9120	0.3449	0.134*	
C79	0.7521 (5)	0.9016 (2)	0.3186 (4)	0.1172 (17)*	
H79A	0.7315	0.8699	0.2715	0.141*	
H79B	0.7635	0.9304	0.2713	0.141*	
C80	0.6510 (6)	0.9131 (3)	0.3584 (6)	0.147 (2)*	
H80A	0.5718	0.9181	0.2938	0.221*	
H80B	0.6711	0.9449	0.4037	0.221*	
H80C	0.6401	0.8845	0.4052	0.221*	
O11	0.8631 (3)	0.89420 (13)	0.4096 (3)	0.1138 (10)*	
C1	0.5785 (3)	0.53367 (10)	0.6920 (2)	0.0518 (7)	
C2	0.5159 (2)	0.59995 (12)	0.9031 (2)	0.0456 (6)	
C3	0.37567 (19)	0.72557 (7)	0.53935 (15)	0.0230 (4)	
C4	0.44781 (18)	0.75555 (7)	0.65556 (16)	0.0248 (4)	
C5	0.4165 (2)	0.73789 (8)	0.43790 (16)	0.0287 (4)	
C6	0.3402 (2)	0.72021 (9)	0.33200 (16)	0.0377 (5)	
H6	0.2633	0.7022	0.3239	0.045*	
C7	0.3749 (2)	0.72856 (10)	0.23819 (19)	0.0507 (7)	
H7	0.3217	0.7161	0.1664	0.061*	
C8	0.4858 (2)	0.75483 (11)	0.24739 (19)	0.0573 (8)	
H8	0.5071	0.7620	0.1821	0.069*	
C9	0.5654 (3)	0.77058 (10)	0.35312 (17)	0.0522 (7)	
H9	0.6438	0.7874	0.3610	0.063*	
C10	0.5319 (2)	0.76214 (9)	0.44750 (19)	0.0375 (5)	
H10	0.5879	0.7729	0.5198	0.045*	
C11	0.23051 (19)	0.72664 (7)	0.50856 (15)	0.0242 (4)	
C12	0.1685 (2)	0.68911 (8)	0.54920 (17)	0.0297 (4)	
H12	0.2158	0.6610	0.5922	0.036*	
C13	0.0377 (2)	0.69246 (9)	0.52739 (19)	0.0346 (5)	
H13	−0.0041	0.6662	0.5544	0.042*	
C14	−0.0318 (2)	0.73353 (9)	0.46688 (18)	0.0347 (5)	
H14	−0.1209	0.7360	0.4534	0.042*	
C15	0.0287 (2)	0.77076 (8)	0.42623 (17)	0.0338 (5)	
H15	−0.0188	0.7992	0.3846	0.041*	
C16	0.1582 (2)	0.76722 (8)	0.44540 (16)	0.0289 (4)	
H16	0.1984	0.7928	0.4151	0.035*	
C17	0.37177 (18)	0.75349 (6)	0.73659 (15)	0.0268 (4)	
C18	0.27163 (19)	0.78775 (8)	0.72410 (17)	0.0316 (4)	
H18	0.2514	0.8130	0.6657	0.038*	
C19	0.2012 (2)	0.78574 (9)	0.79491 (17)	0.0406 (5)	
H19	0.1335	0.8096	0.7850	0.049*	
C20	0.2282 (3)	0.74940 (9)	0.8798 (2)	0.0542 (7)	
H20	0.1775	0.7471	0.9266	0.065*	
C21	0.3298 (3)	0.71647 (12)	0.8955 (2)	0.0654 (9)	
H21	0.3513	0.6921	0.9557	0.078*	
C22	0.4007 (3)	0.71839 (10)	0.82517 (19)	0.0470 (6)	
H22	0.4705	0.6953	0.8375	0.056*	
C23	0.4872 (2)	0.81179 (7)	0.65172 (18)	0.0287 (4)	
C24	0.4264 (2)	0.84704 (7)	0.5669 (2)	0.0407 (5)	
H24	0.3584	0.8356	0.5019	0.049*	
C25	0.4628 (3)	0.89834 (8)	0.5752 (2)	0.0495 (6)	
H25	0.4194	0.9218	0.5163	0.059*	
C26	0.5618 (2)	0.91583 (9)	0.66831 (19)	0.0506 (7)	
H26	0.5870	0.9511	0.6740	0.061*	
C27	0.6238 (2)	0.88133 (7)	0.7529 (2)	0.0505 (7)	
H27	0.6930	0.8928	0.8170	0.061*	
C28	0.5857 (2)	0.83023 (8)	0.7449 (2)	0.0377 (5)	
H28	0.6282	0.8071	0.8048	0.045*	
C29	0.75300 (19)	0.63414 (7)	0.60830 (16)	0.0236 (4)	
C30	0.75663 (16)	0.59539 (10)	0.53180 (14)	0.0268 (3)	
C31	0.8683 (2)	0.56572 (7)	0.56195 (17)	0.0298 (4)	
H31	0.8735	0.5385	0.5130	0.036*	
C32	0.9723 (2)	0.57425 (8)	0.66043 (18)	0.0282 (4)	
C33	0.96708 (19)	0.61368 (7)	0.73284 (16)	0.0253 (4)	
H33	1.0380	0.6202	0.7998	0.030*	
C34	0.85706 (19)	0.64382 (7)	0.70688 (16)	0.0225 (4)	
C35	0.85814 (18)	0.68962 (7)	0.77842 (16)	0.0241 (4)	
C36	0.85984 (19)	0.68565 (7)	0.88844 (16)	0.0240 (4)	
C37	0.8629 (2)	0.72994 (8)	0.95425 (16)	0.0260 (4)	
C38	0.8742 (2)	0.77697 (8)	0.90746 (17)	0.0285 (4)	
H38	0.8807	0.8074	0.9516	0.034*	
C39	0.8764 (2)	0.78185 (7)	0.79794 (18)	0.0277 (4)	
C40	0.86457 (19)	0.73841 (7)	0.73202 (17)	0.0256 (4)	
H40	0.8608	0.7414	0.6557	0.031*	
C41	0.65315 (19)	0.58828 (10)	0.41376 (16)	0.0347 (5)	
C42	0.6587 (4)	0.63533 (12)	0.3432 (2)	0.0581 (8)	
H42A	0.6297	0.6659	0.3735	0.087*	
H42B	0.6031	0.6298	0.2646	0.087*	
H42C	0.7468	0.6406	0.3462	0.087*	
C43	0.5178 (2)	0.58264 (12)	0.4152 (2)	0.0536 (8)	
H43A	0.4934	0.6144	0.4453	0.080*	
H43B	0.5143	0.5533	0.4633	0.080*	
H43C	0.4585	0.5767	0.3381	0.080*	
C44	0.6792 (3)	0.53988 (12)	0.3542 (2)	0.0529 (8)	
H44A	0.6122	0.5363	0.2797	0.079*	
H44B	0.6794	0.5092	0.4000	0.079*	
H44C	0.7625	0.5432	0.3449	0.079*	
C45	1.1894 (2)	0.55561 (10)	0.7650 (2)	0.0428 (5)	
H45A	1.2151	0.5911	0.7556	0.064*	
H45B	1.2572	0.5316	0.7643	0.064*	
H45C	1.1751	0.5526	0.8372	0.064*	
C46	0.8585 (2)	0.72778 (9)	1.07566 (17)	0.0338 (5)	
C47	0.7439 (3)	0.69620 (10)	1.0791 (2)	0.0415 (6)	
H47A	0.7534	0.6602	1.0588	0.062*	
H47B	0.6651	0.7107	1.0252	0.062*	
H47C	0.7399	0.6975	1.1556	0.062*	
C48	0.9848 (3)	0.70463 (10)	1.15472 (19)	0.0438 (6)	
H48A	0.9823	0.7021	1.2316	0.066*	
H48B	1.0560	0.7269	1.1552	0.066*	
H48C	0.9965	0.6701	1.1279	0.066*	
C49	0.8440 (3)	0.78176 (9)	1.11995 (19)	0.0455 (6)	
H49A	0.8371	0.7787	1.1952	0.068*	
H49B	0.7667	0.7983	1.0681	0.068*	
H49C	0.9188	0.8028	1.1249	0.068*	
C50	0.8673 (3)	0.83944 (9)	0.6481 (2)	0.0445 (6)	
H50A	0.9270	0.8189	0.6237	0.067*	
H50B	0.8783	0.8762	0.6349	0.067*	
H50C	0.7794	0.8290	0.6047	0.067*	
C51	0.8631 (2)	0.51759 (7)	1.06368 (16)	0.0272 (4)	
C52	0.9253 (2)	0.50443 (7)	0.96605 (16)	0.0258 (4)	
C53	0.9628 (2)	0.52742 (7)	1.17970 (17)	0.0333 (5)	
C54	0.9590 (3)	0.57220 (9)	1.23876 (18)	0.0436 (6)	
H54	0.8948	0.5974	1.2056	0.052*	
C55	1.0473 (3)	0.58061 (11)	1.3451 (2)	0.0635 (8)	
H55	1.0434	0.6115	1.3845	0.076*	
C56	1.1416 (3)	0.54451 (14)	1.3952 (2)	0.0684 (9)	
H56	1.2029	0.5508	1.4681	0.082*	
C57	1.1457 (3)	0.49955 (12)	1.3383 (2)	0.0553 (7)	
H57	1.2091	0.4742	1.3722	0.066*	
C58	1.0572 (2)	0.49143 (9)	1.23184 (19)	0.0410 (5)	
H58	1.0609	0.4603	1.1930	0.049*	
C59	0.7618 (2)	0.47876 (8)	1.06974 (19)	0.0297 (5)	
C60	0.7693 (2)	0.45389 (8)	1.1700 (2)	0.0407 (5)	
H60	0.8376	0.4619	1.2379	0.049*	
C61	0.6786 (2)	0.41769 (9)	1.1717 (2)	0.0525 (7)	
H61	0.6854	0.4009	1.2406	0.063*	
C62	0.5784 (3)	0.40585 (10)	1.07396 (18)	0.0524 (7)	
H62	0.5181	0.3800	1.0746	0.063*	
C63	0.5666 (2)	0.43203 (9)	0.9752 (2)	0.0488 (6)	
H63	0.4963	0.4250	0.9081	0.059*	
C64	0.6570 (2)	0.46836 (8)	0.9739 (2)	0.0382 (5)	
H64	0.6470	0.4866	0.9059	0.046*	
C65	1.0696 (2)	0.51295 (9)	1.00494 (17)	0.0320 (5)	
C66	1.1212 (2)	0.56104 (10)	1.04461 (18)	0.0391 (5)	
H66	1.0658	0.5888	1.0457	0.047*	
C67	1.25186 (18)	0.56925 (11)	1.0827 (2)	0.0546 (7)	
H67	1.2854	0.6024	1.1102	0.065*	
C68	1.3338 (3)	0.52943 (10)	1.0807 (2)	0.0651 (9)	
H68	1.4237	0.5348	1.1078	0.078*	
C69	1.2832 (2)	0.48177 (11)	1.0386 (2)	0.0635 (9)	
H69	1.3387	0.4544	1.0355	0.076*	
C70	1.1525 (2)	0.47359 (10)	1.0011 (2)	0.0458 (6)	
H70	1.1191	0.4406	0.9724	0.055*	
C71	0.8872 (2)	0.45146 (8)	0.91157 (18)	0.0300 (4)	
C72	0.9014 (2)	0.40673 (8)	0.9771 (2)	0.0355 (5)	
H72	0.9386	0.4089	1.0572	0.043*	
C73	0.8617 (3)	0.35940 (9)	0.9266 (2)	0.0446 (6)	
H73	0.8711	0.3293	0.9722	0.053*	
C74	0.8087 (3)	0.35539 (10)	0.8108 (3)	0.0591 (8)	
H74	0.7785	0.3230	0.7761	0.071*	
C75	0.7999 (4)	0.39903 (10)	0.7457 (2)	0.0620 (8)	
H75	0.7664	0.3964	0.6655	0.074*	
C76	0.8393 (3)	0.44637 (9)	0.79552 (19)	0.0419 (6)	
H76	0.8334	0.4760	0.7492	0.050*	
O1	0.41561 (13)	0.67214 (5)	0.57154 (11)	0.0249 (3)	
O2	0.56444 (13)	0.72564 (5)	0.70404 (11)	0.0258 (3)	
O3	0.64584 (13)	0.66599 (5)	0.58506 (11)	0.0265 (3)	
O4	0.87143 (14)	0.63686 (5)	0.93978 (11)	0.0266 (3)	
O5	0.79305 (14)	0.56579 (5)	1.02459 (11)	0.0285 (3)	
O6	0.86838 (14)	0.54274 (5)	0.87786 (11)	0.0258 (3)	
O7	0.5764 (3)	0.49777 (8)	0.6399 (2)	0.0890 (9)	
O8	0.4804 (2)	0.60351 (9)	0.97680 (18)	0.0691 (6)	
O9	1.07528 (16)	0.54333 (6)	0.67518 (14)	0.0421 (4)	
O10	0.89204 (16)	0.83106 (5)	0.76520 (13)	0.0341 (3)	
P1	0.55672 (5)	0.66565 (2)	0.66426 (4)	0.02305 (10)	
P2	0.78003 (5)	0.586778 (19)	0.90123 (4)	0.02370 (10)	
Rh1	0.574142 (14)	0.594078 (6)	0.777762 (12)	0.02906 (4)	
H1	0.444 (3)	0.5921 (15)	0.708 (2)	0.062 (7)*	

### Atomic displacement parameters (Å^2^)

**Table:**

	*U*^11^	*U*^22^	*U*^33^	*U*^12^	*U*^13^	*U*^23^
C1	0.0549 (18)	0.0355 (13)	0.0473 (15)	−0.0018 (12)	−0.0045 (13)	0.0005 (12)
C2	0.0323 (11)	0.0520 (15)	0.0581 (13)	0.0060 (12)	0.0228 (10)	0.0182 (13)
C3	0.0249 (10)	0.0200 (8)	0.0234 (9)	0.0035 (7)	0.0074 (7)	0.0006 (7)
C4	0.0210 (10)	0.0247 (9)	0.0263 (9)	0.0047 (7)	0.0052 (7)	−0.0008 (7)
C5	0.0339 (12)	0.0267 (9)	0.0273 (10)	0.0069 (8)	0.0129 (9)	0.0036 (8)
C6	0.0387 (13)	0.0452 (13)	0.0293 (11)	0.0082 (10)	0.0117 (9)	0.0003 (9)
C7	0.0610 (18)	0.0641 (17)	0.0280 (11)	0.0204 (14)	0.0166 (11)	0.0042 (11)
C8	0.076 (2)	0.0670 (17)	0.0435 (14)	0.0223 (16)	0.0392 (15)	0.0192 (13)
C9	0.0569 (17)	0.0479 (14)	0.0683 (18)	0.0032 (12)	0.0427 (15)	0.0115 (12)
C10	0.0402 (13)	0.0351 (11)	0.0430 (12)	0.0011 (10)	0.0215 (11)	0.0007 (9)
C11	0.0260 (10)	0.0246 (9)	0.0206 (8)	0.0017 (8)	0.0063 (7)	−0.0038 (7)
C12	0.0292 (11)	0.0289 (10)	0.0298 (10)	0.0006 (8)	0.0086 (8)	−0.0012 (8)
C13	0.0319 (12)	0.0392 (11)	0.0353 (11)	−0.0044 (9)	0.0148 (9)	−0.0018 (9)
C14	0.0250 (11)	0.0433 (12)	0.0345 (11)	0.0029 (9)	0.0084 (9)	−0.0090 (9)
C15	0.0284 (11)	0.0361 (11)	0.0297 (10)	0.0103 (9)	0.0010 (8)	−0.0031 (8)
C16	0.0299 (11)	0.0272 (10)	0.0268 (10)	0.0024 (8)	0.0062 (8)	0.0006 (7)
C17	0.0279 (11)	0.0267 (9)	0.0242 (9)	0.0002 (8)	0.0069 (8)	−0.0060 (7)
C18	0.0301 (11)	0.0311 (10)	0.0324 (11)	0.0047 (9)	0.0092 (9)	−0.0064 (8)
C19	0.0338 (13)	0.0451 (13)	0.0468 (13)	0.0075 (10)	0.0189 (10)	−0.0047 (10)
C20	0.0677 (19)	0.0566 (16)	0.0576 (16)	0.0150 (14)	0.0463 (15)	0.0055 (13)
C21	0.093 (2)	0.0676 (18)	0.0553 (17)	0.0359 (18)	0.0504 (17)	0.0264 (14)
C22	0.0567 (16)	0.0516 (14)	0.0386 (12)	0.0277 (12)	0.0241 (12)	0.0081 (10)
C23	0.0251 (11)	0.0271 (9)	0.0362 (11)	−0.0006 (8)	0.0136 (9)	−0.0079 (8)
C24	0.0422 (14)	0.0289 (10)	0.0471 (13)	−0.0032 (10)	0.0106 (11)	0.0014 (9)
C25	0.0583 (18)	0.0297 (11)	0.0613 (16)	−0.0021 (11)	0.0217 (14)	0.0027 (11)
C26	0.0515 (17)	0.0288 (11)	0.0818 (19)	−0.0114 (11)	0.0359 (15)	−0.0152 (12)
C27	0.0357 (14)	0.0423 (13)	0.0729 (18)	−0.0080 (11)	0.0179 (13)	−0.0277 (13)
C28	0.0309 (12)	0.0329 (11)	0.0449 (12)	0.0017 (9)	0.0076 (10)	−0.0102 (9)
C29	0.0230 (10)	0.0228 (8)	0.0267 (9)	0.0005 (7)	0.0108 (8)	0.0015 (7)
C30	0.0261 (8)	0.0258 (8)	0.0289 (8)	0.0002 (11)	0.0098 (7)	−0.0012 (10)
C31	0.0312 (11)	0.0252 (10)	0.0340 (10)	0.0036 (8)	0.0126 (9)	−0.0061 (8)
C32	0.0262 (11)	0.0260 (9)	0.0347 (10)	0.0041 (8)	0.0135 (8)	0.0000 (8)
C33	0.0219 (10)	0.0274 (8)	0.0265 (9)	−0.0022 (7)	0.0083 (8)	0.0014 (7)
C34	0.0249 (10)	0.0205 (8)	0.0254 (9)	−0.0012 (7)	0.0127 (8)	0.0005 (7)
C35	0.0199 (10)	0.0258 (9)	0.0259 (9)	−0.0003 (7)	0.0070 (7)	−0.0029 (7)
C36	0.0223 (10)	0.0243 (9)	0.0259 (9)	−0.0024 (7)	0.0089 (8)	0.0004 (7)
C37	0.0220 (10)	0.0302 (10)	0.0233 (9)	−0.0022 (8)	0.0045 (8)	−0.0037 (8)
C38	0.0255 (11)	0.0278 (10)	0.0298 (10)	−0.0024 (8)	0.0066 (8)	−0.0080 (8)
C39	0.0271 (11)	0.0231 (9)	0.0341 (10)	−0.0015 (8)	0.0123 (9)	0.0018 (8)
C40	0.0256 (11)	0.0255 (9)	0.0266 (9)	−0.0002 (8)	0.0101 (8)	0.0005 (7)
C41	0.0315 (10)	0.0393 (12)	0.0298 (9)	0.0041 (11)	0.0061 (7)	−0.0084 (10)
C42	0.069 (2)	0.0575 (17)	0.0351 (14)	−0.0011 (15)	0.0024 (13)	−0.0005 (12)
C43	0.0320 (12)	0.081 (2)	0.0413 (12)	−0.0052 (13)	0.0050 (9)	−0.0233 (13)
C44	0.0440 (17)	0.0582 (17)	0.0421 (14)	0.0096 (13)	−0.0032 (12)	−0.0272 (12)
C45	0.0266 (12)	0.0497 (13)	0.0498 (14)	0.0097 (10)	0.0102 (10)	0.0030 (11)
C46	0.0405 (13)	0.0381 (11)	0.0235 (9)	−0.0011 (9)	0.0117 (9)	−0.0047 (8)
C47	0.0504 (16)	0.0441 (14)	0.0369 (13)	−0.0003 (12)	0.0238 (12)	−0.0023 (10)
C48	0.0474 (15)	0.0489 (14)	0.0285 (11)	0.0017 (11)	0.0049 (10)	−0.0038 (10)
C49	0.0662 (18)	0.0410 (12)	0.0310 (11)	0.0029 (12)	0.0191 (11)	−0.0073 (9)
C50	0.0620 (17)	0.0319 (11)	0.0430 (13)	−0.0021 (11)	0.0225 (12)	0.0072 (9)
C51	0.0318 (11)	0.0258 (9)	0.0262 (9)	0.0054 (8)	0.0128 (8)	0.0046 (7)
C52	0.0262 (11)	0.0271 (9)	0.0250 (9)	0.0050 (8)	0.0103 (8)	0.0060 (7)
C53	0.0379 (13)	0.0385 (11)	0.0250 (10)	0.0000 (9)	0.0127 (9)	0.0043 (8)
C54	0.0526 (16)	0.0460 (12)	0.0314 (11)	0.0057 (11)	0.0136 (10)	−0.0014 (9)
C55	0.084 (2)	0.062 (2)	0.0370 (12)	0.0020 (15)	0.0107 (13)	−0.0136 (12)
C56	0.072 (2)	0.089 (2)	0.0295 (13)	0.0022 (18)	−0.0022 (13)	−0.0054 (14)
C57	0.0528 (17)	0.0710 (19)	0.0356 (13)	0.0133 (14)	0.0069 (12)	0.0103 (12)
C58	0.0445 (14)	0.0481 (13)	0.0293 (11)	0.0094 (11)	0.0112 (10)	0.0067 (10)
C59	0.0369 (13)	0.0248 (10)	0.0343 (11)	0.0063 (9)	0.0211 (10)	0.0057 (8)
C60	0.0554 (16)	0.0352 (11)	0.0409 (12)	0.0044 (11)	0.0287 (12)	0.0091 (10)
C61	0.071 (2)	0.0415 (13)	0.0588 (16)	0.0022 (13)	0.0407 (16)	0.0163 (12)
C62	0.0511 (17)	0.0388 (13)	0.081 (2)	−0.0036 (12)	0.0399 (16)	0.0117 (13)
C63	0.0361 (14)	0.0475 (14)	0.0645 (17)	−0.0049 (11)	0.0196 (13)	0.0049 (12)
C64	0.0335 (13)	0.0384 (12)	0.0461 (13)	0.0018 (10)	0.0180 (11)	0.0111 (10)
C65	0.0288 (11)	0.0412 (12)	0.0262 (10)	0.0039 (9)	0.0098 (8)	0.0104 (8)
C66	0.0323 (12)	0.0510 (13)	0.0320 (11)	−0.0076 (10)	0.0084 (9)	0.0029 (10)
C67	0.0355 (14)	0.0747 (18)	0.0444 (14)	−0.0131 (13)	0.0023 (11)	0.0148 (13)
C68	0.0260 (14)	0.100 (2)	0.0613 (17)	−0.0023 (15)	0.0052 (12)	0.0395 (17)
C69	0.0347 (15)	0.081 (2)	0.081 (2)	0.0246 (15)	0.0285 (15)	0.0445 (17)
C70	0.0333 (13)	0.0500 (14)	0.0572 (15)	0.0129 (11)	0.0196 (11)	0.0210 (12)
C71	0.0294 (12)	0.0295 (10)	0.0356 (11)	0.0037 (8)	0.0170 (9)	0.0009 (8)
C72	0.0394 (13)	0.0304 (11)	0.0401 (12)	0.0096 (9)	0.0180 (10)	0.0074 (9)
C73	0.0563 (16)	0.0260 (10)	0.0588 (15)	0.0044 (10)	0.0294 (13)	0.0051 (10)
C74	0.083 (2)	0.0314 (12)	0.0650 (18)	−0.0071 (13)	0.0281 (17)	−0.0115 (12)
C75	0.096 (3)	0.0431 (14)	0.0418 (14)	−0.0058 (15)	0.0176 (16)	−0.0098 (11)
C76	0.0628 (17)	0.0309 (11)	0.0324 (11)	0.0014 (11)	0.0169 (11)	0.0003 (9)
O1	0.0233 (7)	0.0216 (6)	0.0277 (7)	0.0013 (5)	0.0060 (5)	−0.0010 (5)
O2	0.0208 (7)	0.0273 (7)	0.0261 (6)	0.0041 (5)	0.0041 (5)	−0.0031 (5)
O3	0.0251 (7)	0.0269 (6)	0.0270 (7)	0.0056 (6)	0.0084 (6)	−0.0006 (5)
O4	0.0296 (8)	0.0247 (7)	0.0222 (7)	−0.0006 (6)	0.0047 (6)	0.0021 (5)
O5	0.0336 (8)	0.0256 (7)	0.0291 (7)	0.0061 (6)	0.0145 (6)	0.0048 (5)
O6	0.0300 (8)	0.0251 (7)	0.0225 (6)	0.0036 (6)	0.0092 (6)	0.0038 (5)
O7	0.115 (2)	0.0415 (11)	0.0759 (15)	0.0036 (13)	−0.0104 (14)	−0.0194 (11)
O8	0.0672 (13)	0.0842 (17)	0.0769 (13)	0.0133 (12)	0.0514 (11)	0.0264 (12)
O9	0.0313 (9)	0.0427 (9)	0.0463 (9)	0.0127 (7)	0.0056 (7)	−0.0105 (7)
O10	0.0423 (9)	0.0219 (7)	0.0406 (8)	−0.0021 (6)	0.0176 (7)	0.0014 (6)
P1	0.0218 (3)	0.0234 (2)	0.0234 (2)	0.0023 (2)	0.0071 (2)	0.00024 (19)
P2	0.0243 (2)	0.0229 (3)	0.0240 (2)	0.0010 (2)	0.00854 (17)	0.00250 (18)
Rh1	0.02251 (7)	0.02832 (7)	0.03357 (7)	−0.00073 (8)	0.00617 (5)	0.00755 (8)

### Geometric parameters (Å, º)

**Table:**

C77—C78	1.414 (5)	C41—C42	1.520 (4)
C77—H77A	0.9800	C41—C43	1.523 (3)
C77—H77B	0.9800	C41—C44	1.537 (3)
C77—H77C	0.9800	C42—H42A	0.9800
C78—O11	1.369 (4)	C42—H42B	0.9800
C78—H78A	0.9900	C42—H42C	0.9800
C78—H78B	0.9900	C43—H43A	0.9800
C79—O11	1.377 (4)	C43—H43B	0.9800
C79—C80	1.415 (5)	C43—H43C	0.9800
C79—H79A	0.9900	C44—H44A	0.9800
C79—H79B	0.9900	C44—H44B	0.9800
C80—H80A	0.9800	C44—H44C	0.9800
C80—H80B	0.9800	C45—O9	1.415 (3)
C80—H80C	0.9800	C45—H45A	0.9800
C1—O7	1.132 (3)	C45—H45B	0.9800
C1—Rh1	1.909 (3)	C45—H45C	0.9800
C2—O8	1.133 (3)	C46—C47	1.529 (4)
C2—Rh1	1.909 (2)	C46—C49	1.532 (3)
C3—O1	1.464 (2)	C46—C48	1.536 (3)
C3—C11	1.528 (3)	C47—H47A	0.9800
C3—C5	1.531 (3)	C47—H47B	0.9800
C3—C4	1.604 (3)	C47—H47C	0.9800
C4—O2	1.453 (2)	C48—H48A	0.9800
C4—C23	1.524 (3)	C48—H48B	0.9800
C4—C17	1.537 (3)	C48—H48C	0.9800
C5—C6	1.388 (3)	C49—H49A	0.9800
C5—C10	1.398 (3)	C49—H49B	0.9800
C6—C7	1.3814 (18)	C49—H49C	0.9800
C6—H6	0.9500	C50—O10	1.421 (3)
C7—C8	1.3797 (19)	C50—H50A	0.9800
C7—H7	0.9500	C50—H50B	0.9800
C8—C9	1.3811 (19)	C50—H50C	0.9800
C8—H8	0.9500	C51—O5	1.462 (2)
C9—C10	1.3811 (18)	C51—C53	1.520 (3)
C9—H9	0.9500	C51—C59	1.532 (3)
C10—H10	0.9500	C51—C52	1.643 (3)
C11—C12	1.387 (3)	C52—O6	1.459 (2)
C11—C16	1.392 (3)	C52—C71	1.524 (3)
C12—C13	1.390 (3)	C52—C65	1.527 (3)
C12—H12	0.9500	C53—C54	1.385 (2)
C13—C14	1.378 (3)	C53—C58	1.387 (2)
C13—H13	0.9500	C54—C55	1.377 (4)
C14—C15	1.372 (3)	C54—H54	0.9500
C14—H14	0.9500	C55—C56	1.384 (4)
C15—C16	1.382 (3)	C55—H55	0.9500
C15—H15	0.9500	C56—C57	1.374 (4)
C16—H16	0.9500	C56—H56	0.9500
C17—C22	1.386 (2)	C57—C58	1.378 (4)
C17—C18	1.391 (2)	C57—H57	0.9500
C18—C19	1.3779 (18)	C58—H58	0.9500
C18—H18	0.9500	C59—C64	1.385 (3)
C19—C20	1.3754 (18)	C59—C60	1.393 (3)
C19—H19	0.9500	C60—C61	1.3835 (19)
C20—C21	1.3751 (18)	C60—H60	0.9500
C20—H20	0.9500	C61—C62	1.3805 (19)
C21—C22	1.3778 (18)	C61—H61	0.9500
C21—H21	0.9500	C62—C63	1.3817 (19)
C22—H22	0.9500	C62—H62	0.9500
C23—C28	1.385 (3)	C63—C64	1.3814 (19)
C23—C24	1.389 (3)	C63—H63	0.9500
C24—C25	1.3800 (18)	C64—H64	0.9500
C24—H24	0.9500	C65—C70	1.386 (3)
C25—C26	1.3791 (18)	C65—C66	1.388 (3)
C25—H25	0.9500	C66—C67	1.3835 (19)
C26—C27	1.3775 (18)	C66—H66	0.9500
C26—H26	0.9500	C67—C68	1.3823 (19)
C27—C28	1.3802 (18)	C67—H67	0.9500
C27—H27	0.9500	C68—C69	1.382 (2)
C28—H28	0.9500	C68—H68	0.9500
C29—O3	1.396 (2)	C69—C70	1.3840 (19)
C29—C34	1.397 (3)	C69—H69	0.9500
C29—C30	1.400 (3)	C70—H70	0.9500
C30—C31	1.398 (3)	C71—C76	1.378 (3)
C30—C41	1.545 (2)	C71—C72	1.397 (3)
C31—C32	1.391 (3)	C72—C73	1.379 (3)
C31—H31	0.9500	C72—H72	0.9500
C32—O9	1.358 (2)	C73—C74	1.375 (4)
C32—C33	1.382 (3)	C73—H73	0.9500
C33—C34	1.393 (3)	C74—C75	1.378 (4)
C33—H33	0.9500	C74—H74	0.9500
C34—C35	1.485 (3)	C75—C76	1.376 (3)
C35—C36	1.383 (3)	C75—H75	0.9500
C35—C40	1.402 (3)	C76—H76	0.9500
C36—O4	1.403 (2)	O1—P1	1.6106 (14)
C36—C37	1.407 (3)	O2—P1	1.6224 (14)
C37—C38	1.375 (3)	O3—P1	1.6336 (14)
C37—C46	1.548 (3)	O4—P2	1.6146 (14)
C38—C39	1.394 (3)	O5—P2	1.6039 (14)
C38—H38	0.9500	O6—P2	1.5979 (14)
C39—O10	1.367 (2)	P1—Rh1	2.3045 (5)
C39—C40	1.375 (3)	P2—Rh1	2.2913 (5)
C40—H40	0.9500	Rh1—H1	1.42 (3)
			
C78—C77—H77A	109.5	H43A—C43—H43B	109.5
C78—C77—H77B	109.5	C41—C43—H43C	109.5
H77A—C77—H77B	109.5	H43A—C43—H43C	109.5
C78—C77—H77C	109.5	H43B—C43—H43C	109.5
H77A—C77—H77C	109.5	C41—C44—H44A	109.5
H77B—C77—H77C	109.5	C41—C44—H44B	109.5
O11—C78—C77	116.2 (4)	H44A—C44—H44B	109.5
O11—C78—H78A	108.2	C41—C44—H44C	109.5
C77—C78—H78A	108.2	H44A—C44—H44C	109.5
O11—C78—H78B	108.2	H44B—C44—H44C	109.5
C77—C78—H78B	108.2	O9—C45—H45A	109.5
H78A—C78—H78B	107.4	O9—C45—H45B	109.5
O11—C79—C80	109.3 (5)	H45A—C45—H45B	109.5
O11—C79—H79A	109.8	O9—C45—H45C	109.5
C80—C79—H79A	109.8	H45A—C45—H45C	109.5
O11—C79—H79B	109.8	H45B—C45—H45C	109.5
C80—C79—H79B	109.8	C47—C46—C49	106.3 (2)
H79A—C79—H79B	108.3	C47—C46—C48	111.1 (2)
C79—C80—H80A	109.5	C49—C46—C48	107.92 (19)
C79—C80—H80B	109.5	C47—C46—C37	111.10 (18)
H80A—C80—H80B	109.5	C49—C46—C37	111.75 (18)
C79—C80—H80C	109.5	C48—C46—C37	108.60 (18)
H80A—C80—H80C	109.5	C46—C47—H47A	109.5
H80B—C80—H80C	109.5	C46—C47—H47B	109.5
C78—O11—C79	118.4 (4)	H47A—C47—H47B	109.5
O7—C1—Rh1	177.5 (3)	C46—C47—H47C	109.5
O8—C2—Rh1	179.4 (2)	H47A—C47—H47C	109.5
O1—C3—C11	106.33 (15)	H47B—C47—H47C	109.5
O1—C3—C5	106.10 (14)	C46—C48—H48A	109.5
C11—C3—C5	112.30 (15)	C46—C48—H48B	109.5
O1—C3—C4	101.22 (13)	H48A—C48—H48B	109.5
C11—C3—C4	112.02 (15)	C46—C48—H48C	109.5
C5—C3—C4	117.43 (16)	H48A—C48—H48C	109.5
O2—C4—C23	107.17 (16)	H48B—C48—H48C	109.5
O2—C4—C17	108.77 (14)	C46—C49—H49A	109.5
C23—C4—C17	106.57 (15)	C46—C49—H49B	109.5
O2—C4—C3	102.67 (14)	H49A—C49—H49B	109.5
C23—C4—C3	119.38 (16)	C46—C49—H49C	109.5
C17—C4—C3	111.80 (16)	H49A—C49—H49C	109.5
C6—C5—C10	118.05 (19)	H49B—C49—H49C	109.5
C6—C5—C3	118.22 (19)	O10—C50—H50A	109.5
C10—C5—C3	123.49 (18)	O10—C50—H50B	109.5
C7—C6—C5	120.7 (2)	H50A—C50—H50B	109.5
C7—C6—H6	119.6	O10—C50—H50C	109.5
C5—C6—H6	119.6	H50A—C50—H50C	109.5
C8—C7—C6	120.9 (2)	H50B—C50—H50C	109.5
C8—C7—H7	119.6	O5—C51—C53	107.98 (15)
C6—C7—H7	119.6	O5—C51—C59	104.95 (16)
C7—C8—C9	118.9 (2)	C53—C51—C59	111.58 (17)
C7—C8—H8	120.5	O5—C51—C52	103.82 (14)
C9—C8—H8	120.5	C53—C51—C52	113.35 (17)
C10—C9—C8	120.6 (2)	C59—C51—C52	114.28 (16)
C10—C9—H9	119.7	O6—C52—C71	106.68 (16)
C8—C9—H9	119.7	O6—C52—C65	106.17 (15)
C9—C10—C5	120.7 (2)	C71—C52—C65	111.83 (17)
C9—C10—H10	119.6	O6—C52—C51	104.14 (14)
C5—C10—H10	119.6	C71—C52—C51	113.51 (16)
C12—C11—C16	118.27 (19)	C65—C52—C51	113.65 (16)
C12—C11—C3	120.83 (17)	C54—C53—C58	117.7 (2)
C16—C11—C3	120.76 (17)	C54—C53—C51	120.52 (18)
C11—C12—C13	120.4 (2)	C58—C53—C51	121.69 (17)
C11—C12—H12	119.8	C55—C54—C53	120.6 (2)
C13—C12—H12	119.8	C55—C54—H54	119.7
C14—C13—C12	120.5 (2)	C53—C54—H54	119.7
C14—C13—H13	119.7	C54—C55—C56	120.8 (2)
C12—C13—H13	119.7	C54—C55—H55	119.6
C15—C14—C13	119.4 (2)	C56—C55—H55	119.6
C15—C14—H14	120.3	C57—C56—C55	119.3 (3)
C13—C14—H14	120.3	C57—C56—H56	120.3
C14—C15—C16	120.5 (2)	C55—C56—H56	120.3
C14—C15—H15	119.8	C56—C57—C58	119.6 (3)
C16—C15—H15	119.8	C56—C57—H57	120.2
C15—C16—C11	120.9 (2)	C58—C57—H57	120.2
C15—C16—H16	119.6	C57—C58—C53	121.9 (2)
C11—C16—H16	119.6	C57—C58—H58	119.0
C22—C17—C18	117.48 (19)	C53—C58—H58	119.0
C22—C17—C4	121.93 (16)	C64—C59—C60	117.9 (2)
C18—C17—C4	120.58 (15)	C64—C59—C51	119.84 (18)
C19—C18—C17	121.18 (18)	C60—C59—C51	122.2 (2)
C19—C18—H18	119.4	C61—C60—C59	120.8 (2)
C17—C18—H18	119.4	C61—C60—H60	119.6
C20—C19—C18	120.6 (2)	C59—C60—H60	119.6
C20—C19—H19	119.7	C62—C61—C60	120.4 (2)
C18—C19—H19	119.7	C62—C61—H61	119.8
C21—C20—C19	118.8 (2)	C60—C61—H61	119.8
C21—C20—H20	120.6	C61—C62—C63	119.3 (2)
C19—C20—H20	120.6	C61—C62—H62	120.4
C20—C21—C22	120.8 (2)	C63—C62—H62	120.4
C20—C21—H21	119.6	C64—C63—C62	120.1 (2)
C22—C21—H21	119.6	C64—C63—H63	120.0
C21—C22—C17	121.1 (2)	C62—C63—H63	120.0
C21—C22—H22	119.5	C63—C64—C59	121.3 (2)
C17—C22—H22	119.5	C63—C64—H64	119.3
C28—C23—C24	117.27 (19)	C59—C64—H64	119.3
C28—C23—C4	117.01 (18)	C70—C65—C66	118.1 (2)
C24—C23—C4	125.52 (18)	C70—C65—C52	121.6 (2)
C25—C24—C23	121.3 (2)	C66—C65—C52	120.26 (19)
C25—C24—H24	119.3	C67—C66—C65	121.2 (2)
C23—C24—H24	119.3	C67—C66—H66	119.4
C26—C25—C24	120.5 (2)	C65—C66—H66	119.4
C26—C25—H25	119.8	C68—C67—C66	120.2 (3)
C24—C25—H25	119.8	C68—C67—H67	119.9
C27—C26—C25	119.0 (2)	C66—C67—H67	119.9
C27—C26—H26	120.5	C69—C68—C67	119.1 (2)
C25—C26—H26	120.5	C69—C68—H68	120.5
C26—C27—C28	120.2 (2)	C67—C68—H68	120.5
C26—C27—H27	119.9	C68—C69—C70	120.6 (3)
C28—C27—H27	119.9	C68—C69—H69	119.7
C27—C28—C23	121.7 (2)	C70—C69—H69	119.7
C27—C28—H28	119.2	C69—C70—C65	120.8 (2)
C23—C28—H28	119.2	C69—C70—H70	119.6
O3—C29—C34	117.88 (16)	C65—C70—H70	119.6
O3—C29—C30	120.28 (16)	C76—C71—C72	118.1 (2)
C34—C29—C30	121.78 (17)	C76—C71—C52	120.57 (19)
C31—C30—C29	116.15 (16)	C72—C71—C52	121.4 (2)
C31—C30—C41	119.61 (18)	C73—C72—C71	120.6 (2)
C29—C30—C41	123.87 (18)	C73—C72—H72	119.7
C32—C31—C30	122.95 (18)	C71—C72—H72	119.7
C32—C31—H31	118.5	C74—C73—C72	120.5 (2)
C30—C31—H31	118.5	C74—C73—H73	119.8
O9—C32—C33	124.77 (19)	C72—C73—H73	119.8
O9—C32—C31	115.64 (17)	C73—C74—C75	119.0 (2)
C33—C32—C31	119.53 (18)	C73—C74—H74	120.5
C32—C33—C34	119.48 (18)	C75—C74—H74	120.5
C32—C33—H33	120.3	C76—C75—C74	120.7 (3)
C34—C33—H33	120.3	C76—C75—H75	119.6
C33—C34—C29	120.07 (17)	C74—C75—H75	119.6
C33—C34—C35	118.81 (17)	C75—C76—C71	120.9 (2)
C29—C34—C35	120.77 (17)	C75—C76—H76	119.5
C36—C35—C40	119.96 (17)	C71—C76—H76	119.5
C36—C35—C34	122.90 (17)	C3—O1—P1	115.21 (11)
C40—C35—C34	117.05 (17)	C4—O2—P1	115.76 (11)
C35—C36—O4	119.71 (16)	C29—O3—P1	121.75 (12)
C35—C36—C37	121.26 (18)	C36—O4—P2	129.38 (13)
O4—C36—C37	118.72 (17)	C51—O5—P2	118.22 (12)
C38—C37—C36	117.00 (18)	C52—O6—P2	118.46 (12)
C38—C37—C46	119.60 (18)	C32—O9—C45	117.47 (17)
C36—C37—C46	123.37 (18)	C39—O10—C50	117.12 (16)
C37—C38—C39	122.65 (18)	O1—P1—O2	93.35 (7)
C37—C38—H38	118.7	O1—P1—O3	101.81 (7)
C39—C38—H38	118.7	O2—P1—O3	101.83 (7)
O10—C39—C40	124.98 (18)	O1—P1—Rh1	112.62 (5)
O10—C39—C38	115.50 (17)	O2—P1—Rh1	126.26 (5)
C40—C39—C38	119.52 (18)	O3—P1—Rh1	116.40 (5)
C39—C40—C35	119.39 (18)	O6—P2—O5	94.75 (7)
C39—C40—H40	120.3	O6—P2—O4	105.15 (8)
C35—C40—H40	120.3	O5—P2—O4	98.27 (7)
C42—C41—C43	109.0 (2)	O6—P2—Rh1	118.05 (6)
C42—C41—C44	108.3 (2)	O5—P2—Rh1	114.74 (6)
C43—C41—C44	106.5 (2)	O4—P2—Rh1	121.17 (5)
C42—C41—C30	107.4 (2)	C1—Rh1—C2	127.99 (13)
C43—C41—C30	114.37 (17)	C1—Rh1—P2	95.80 (9)
C44—C41—C30	111.23 (19)	C2—Rh1—P2	89.60 (7)
C41—C42—H42A	109.5	C1—Rh1—P1	108.58 (9)
C41—C42—H42B	109.5	C2—Rh1—P1	117.99 (9)
H42A—C42—H42B	109.5	P2—Rh1—P1	109.662 (19)
C41—C42—H42C	109.5	C1—Rh1—H1	80.9 (14)
H42A—C42—H42C	109.5	C2—Rh1—H1	86.9 (10)
H42B—C42—H42C	109.5	P2—Rh1—H1	172.1 (14)
C41—C43—H43A	109.5	P1—Rh1—H1	78.3 (14)
C41—C43—H43B	109.5		
